# Optimization of Distributed Energy Resources Operation in Green Buildings Environment

**DOI:** 10.3390/s24144742

**Published:** 2024-07-22

**Authors:** Safdar Ali, Khizar Hayat, Ibrar Hussain, Ahmad Khan, Dohyeun Kim

**Affiliations:** 1 Department of Software Engineering, The University of Lahore, Main Campus, Lahore 54590, Pakistan; safdar.ali@se.uol.edu.pk (S.A.); khizar.hayat@se.uol.edu.pk (K.H.); ibrar.hussain@cs.uol.edu.pk (I.H.); 2 Faculty of Engineering and Information Technology, Shinawatra University, Bangtoey Samkhok, Pathum Thani 12160, Thailand; 3 Department of Computer Science, COMSATS University Islamabad—Abbottabad Campus, Abbottabad 22060, Pakistan; ahmadkhan@cuiatd.edu.pk; 4 Department of Computer Engineering, Jeju National University, Jeju-si 63243, Jeju-do, Republic of Korea

**Keywords:** energy management, evolutionary algorithms, green buildings, occupants comfort index, energy resources, prediction

## Abstract

Without a well-defined energy management plan, achieving meaningful improvements in human lifestyle becomes challenging. Adequate energy resources are essential for development, but they are both limited and costly. In the literature, several solutions have been proposed for energy management but they either minimize energy consumption or improve the occupant’s comfort index. The energy management problem is a multi-objective problem where the user wants to reduce energy consumption while keeping the occupant’s comfort index intact. To address the multi-objective problem this paper proposed an energy control system for a green environment called PMC (Power Management and Control). The system is based on hybrid energy optimization, energy prediction, and multi-preprocessing. The combination of GA (Genetic Algorithm) and PSO (Particle Swarm Optimization) is performed to make a fusion methodology to improve the occupant comfort index (OCI) and decrease energy utilization. The proposed framework gives a better OCI when compared with its counterparts, the Ant Bee Colony Knowledge Base framework (ABCKB), GA-based prediction framework (GAP), Hybrid Prediction with Single Optimization framework (SOHP), and PSO-based power consumption framework. Compared with the existing AEO framework, the PMC gives practically the same OCI but consumes less energy. The PMC framework additionally accomplished the ideal OCI (i-e 1) when compared with the existing model, FA–GA (i-e 0.98). The PMC model consumed less energy as compared to existing models such as the ABCKB, GAP, PSO, and AEO. The PMC model consumed a little bit more energy than the SOHP but provided a better OCI. The comparative outcomes show the capability of the PMC framework to reduce energy utilization and improve the OCI. Unlike other existing methodologies except for the AEO framework, the PMC technique is additionally confirmed through a simulation by controlling the indoor environment using actuators, such as fan, light, AC, and boiler.

## 1. Introduction

Buildings contribute around 40% of global energy consumption and account for 28% of greenhouse gas (GHG) emissions [1]. Efforts worldwide aim to identify sustainable practices while maintaining the OCI. However, with the global population surpassing eight billion in 2022 [2], innovative solutions are essential to improve living conditions without harming the environment or future energy generations. People spend approximately 90% of their time indoors [3], significantly impacting comfort, quality of life, and energy use. To enhance the OCI perception, indoor environmental quality (IEQ) must be optimized, considering its close link to health, productivity, and social interactions [4]. Despite energy efficiency trends, demand for building active systems persists, emphasizing the need for a pleasant-sounding balance between energy consumption and comfort [5]. Collaboration among designers, architects, engineers, and construction managers is crucial to creating a healthier, sustainable, and energy-efficient indoor environment.

The two prime design goals in the upcoming smart building sector are the user’s comfort and energy management. The fundamental reason is that power consumption utilization rises every day due to enormous growth in domestic applications, while its sources of generation are restricted and expensive. The customer plans to utilize minimum power without compromising the OCI, which constitutes lighting, air quality, and temperature. The importance of low power utilization without disturbing the user’s comfort is an attractive issue for researchers. This creates a big trade-off between power utilization and user comfort [6,7,8,9,10,11,12,13,14,15]. To attain this trade-off, a smart, prevailing, and enhanced power control approach is needed to retain power usage and the OCI at a satisfactory level.

In inhabited sustainable buildings, the important constraints that constitute customers’ quality of life are air quality, thermal comfort, and visual comfort [16].

Temperature refers to the indoor warm and cooling air comfort of the users in smart residential buildings. The cooling and heating scheme is practiced to preserve the hotness in the smart building’s comfort zone. The illumination index is considered to identify the visual comfort of the users in smart and sustainable buildings [17]. The electrical lighting scheme is used to provide visual comfort in energy-efficient smart buildings. CO_2_ concentration is considered a measure of an index to quantify the air quality in residential and smart buildings. The ventilation system is considered to preserve low CO_2_ absorption [18]. Proper arrangement of the environmental parameters serves as the user’s comfort in smart and sustainable residential buildings. We selected the three environmental parameters to assess the user’s comfort and energy efficiency in smart and sustainable residential buildings.

This paper presents the PMC model, an improved, optimized power consumption and prediction framework, which is based on the combination of the PSO and GA for OCI and power saving, in conjunction with the application of an emulator to adjust the indoor environment. The proposed PMC model is intelligent and addresses both energy saving and OCI at the same time along with control of the indoor environment. All the existing studies discussed above, apart from [6,7,8,9,10,11,12,13,14,15,19], either addressed the consumer’s comfort or the building utilizing minimum power, but did not control both at the same time.

The proposed PMC model is designed in such a way as to achieve better results in terms of its defined goals (energy consumption reduction without compromising users’ OCI). The PMC model improves the results significantly compared to the existing, state-of-the-art models. If we talk about the design and significance of the proposed architecture, then the main component in the proposed model and the previous AEO model is the optimization component. The proposed PMC framework optimizes the input parameters much better than the existing AEO framework. The reason behind this is that, in the previous AEO framework, the input data are optimized simultaneously by both the PSO algorithm and GA, while in the proposed PMC framework, the input data are first optimized by the PSO algorithm and then its output parameters are given to the GA to refine the selected individuals further and give the resultant optimized output. That is why the proposed PMC framework optimizes the parameters much better and provides optimized values to the next component in the architecture.

The second point where the proposed model shows its significance is energy consumption. The proposed PMC framework consumed less power in the case of temperature consumption. Additionally, in the case of illumination, the proposed PMC framework devoured less power. Similarly, regarding power utilization in the event of air quality, the proposed PMC framework devoured less power. The total power devoured by the proposed PMC framework is substantially less. This is a significant achievement of the proposed PMC model in terms of power consumption compared to the state-of-the-art frameworks.

The third point where the PMC model improves itself is the OCI. During the time of power failure, the OCI of the PMC model is not disturbed and achieved a high level (i.e., 1). This implies that the proposed PMC model decreased energy utilization without compromising users’ OCI, which is the fundamental objective set and accomplished by the PMC framework.

## 2. Related Work

In the past, uncountable mechanisms and tools in the field of energy efficiency for ecological buildings and some well-known, optimized energy-proficient procedures have been presented for inhabited smart buildings. Frameworks dependent on conventional control issues have been introduced in previous works [20,21,22]. These state-of-the-art controllers include Proportional Integral Derivative (PID) controllers, optimal energy controllers, and adaptive energy controllers. The designer used these controllers to overwhelm the overpass of the temperature. There are some genuine imperfections in these controllers. For example, these controllers need a model of the building under consideration, and these controllers are not user-friendly, as occupants are not involved in choosing the occupant’s comfort index. Different troubles that were observed are checking and observing the parameters that are brought about by nonlinear developments. The control of the interior environment of the building at different zone levels has been presented in [23]. The controller is enhanced and the tenant’s tendencies are seen through a smart card segment.

In the comparative issue area, different control applications frameworks are offered which depend on climate assessment [19,24]. The climate assessment has been judicious in the warming, ventilating, and cooling framework. Another plan, which depends on a multi-specialist control strategy with data integration, has been introduced in [25]. The multi-specialist-based energy productive framework presents a building interior power and comfort conservation approach built on data merging using order-weighted averaging (OWA) accumulation. The multi-specialist-based model accomplished an extraordinary degree of comfort with the least energy usage.

A few individual, public, and building features effectively affect clear comfort in office areas. The association amongst these variables is especially diverse, so to improve comprehension of the relationship between these elements, a model was introduced in [26]. A multi-environmental approach is introduced in [27] which is appropriate for a particular region of the building as well as in the entire building. The approach emphasizes the OCI, giving an improved building climate. The approach additionally allows the assessment of both energy utilization and contaminating effects and considers occupants’ comfort in indoor and open-air climates. Artificial neural networks (ANNs) have been presented to streamline and control the power consumption [28]. The neural organization-based framework affirms power efficiency and gives better maneuvers of solar-, wind-, and hydrogen-energy-based hybrid sustainable independent construction. The GA is applied for power effectiveness and saving in various ways. For example, the GA is implemented for warming, ventilation, and cooling issues [29]. The same technique was used on the control issues of power frameworks, comprising power modules, warm capacity, and warmth siphons [30].

Energy-conservation-estimating strategies are classified into two curricula. One represents time-series-information-gauging frameworks, and the second represents power assessment procedures based on an ANN. The ANN’s framework, which has a magnificent strength and inaccuracy resistance is a compelling framework to take care of complex nonlinear issues. The ANN has been considered by analysts because of its unmistakable model and great presentation in taking care of nonlinear issues; however, it is difficult to set up an efficient framework for every building.

Formerly, the ANN has been implemented for energy prediction in indoor environments [31,32,33,34,35,36,37,38]. These mechanisms often put on a neural organization approach, which encompasses numerous parameters. These parameters are consistently decided by knowledge, and the framework turns out to be hard to set up [39]. That is why it is hard to make a framework which is based on the concept of an ANN. Besides, it has also been observed that, although the neural network (NN) offers little inaccuracy throughout training, the inaccuracy for testing is normally of a bigger order [40]. At the end of the day, when this procedure is applied in real-world scenarios, the forecast precision is not adequate. In addition, the algorithm is expected to change the characters of the multitude of issues into mathematical numbers and modify every interpretation into mathematical computation. There is no doubt that it causes the deficiency of some data, which affects the precision of the prediction. Even though ANN-based price-guessing strategies can likewise be utilized for power estimating, its drawbacks for price estimating argued above confine its supplementary implementation for energy forecasting.

A procedure dependent on the Hidden Markov Model (HMM) is introduced for estimating energy utilization [41]. This technique performs well when contrasted with fundamental forecast frameworks like classification and regression trees, support vector machine, and ANNs; however, the selection of the best HMM is a tedious cycle.

Fixed time series methodologies, for example, autoregressive (AR) [42], Dynamic Regression (DR), Transfer Function (TF) [43], and non-fixed time series frameworks like Autoregressive Integrated Moving Average (ARIMA) [44], have previously been contrived to predict power costs. These techniques can be carried out for energy estimation as well. A few techniques integrate ANN and autoregressive models to foresee the thermal comportment of business offices [45]. A procedure dependent on autoregressive models with exogenous input is proposed to anticipate a one-hour-ahead building energy load [46]. In most recent energy arcades, the series of energy depicts the accompanying features: high recurrence, non-consistent mean and fluctuation, day by day and week after week, month to month, irregularity, schedule impact on the end of the week and public occasions, high unpredictability, and high level of uncommon energy usage. It is difficult to estimate energy precisely; therefore; it requires special supervision in the event of assessing energy changes. Table 1 shows the notations and their meanings used in this paper.

In the same problem area, some evolutionary procedures are also being utilized for assessment in various fields of study which can also be used for energy forecasting [47,48,49,50,51,52,53,54,55]. For example, a strategy called the sunflower optimization technique is utilized for the forecasting of a circuit-based model known as a proton exchange membrane fuel cell (PEMFC) [47]. The model is utilized to lessen the inaccuracy of the squared mistake of an anticipated and genuine yield voltage. Another strategy proposed to decrease the amount of the squared mistake for PEMFC is introduced in [48]. The two models accomplished satisfactory outcomes and limited the hole between real parameters and anticipated parameters. A hybrid framework for the optimization of cost is introduced in [49]. The approach accomplished better outcomes when contrasted with existing philosophies. A multi-target optimization technique for the heat siphon issue is proposed in [50]. The model delivers better outcomes.

## 3. Hybrid Green Energy Efficient System Model

**Figure 1:**
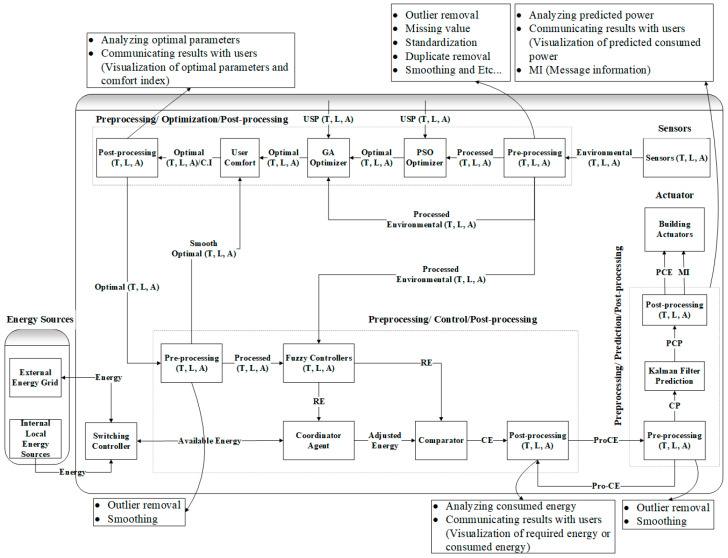
Hybrid energy optimization model.

In Figure 1, we show the proposed improved energy-efficient system model for energy conservation and proficiency in green building environments. Primarily, the real input parameters are handed over to the smoothing segment for preprocessing. Afterward, the smoothed parameters and user-set parameters are passed to the PSO-based optimization part of the system model to develop the optimal parameters (OP). The enhanced Ops are once more passed to the GA segment to further improve the OP. The enhanced OPs are utilized as the OCI to assess the green buildings’ indoor environment. The reason behind optimizing the parameters twice is to obtain a much better comfort index with minimum energy consumption. Then, the post-processing is carried out to disseminate, analyze, and share the outcomes with the consumers. At this point, the OPs are once more preprocessed before they can be progressed to the control component. The fundamental objective of smoothing and preprocessing at this level is to additionally work on the OP and OCI. The OCI is determined yet again by utilizing the refreshed OP. This further develops and improves the occupant’s OCI.

Three fuzzy-dependent controllers are used to update the indoor illumination, air quality, and temperature. Each fuzzy regulator takes as input, the error difference between smoothed environmental parameters and refreshed OP.

The agent-based coordinator fine-tuned the energy based on optimal and required power from the fuzzy-based regulators and existing power from the external energy grid source or inside residential energy sources. The agent-based controller performs the duty of coordinator between the fuzzy regulators and the available power of the building. The devoured power is again post-processed, and the results are distributed and shared with the consumers.

The indoor consumed power (CP) is then preprocessed and forwarded to the forecast segment. Smoothing is performed again at this stage to eradicate any lasting exceptions. In the wake of preprocessing, the CP is refreshed with amended and smoothed power usage. At this point, the refreshed and updated CP is forwarded to the Kalman filter component to foresee energy usage. Towards the end, the post-processing technique is again applied to the predicted power to examine the outcomes and share these outcomes with the consumers. The building actuators obtained the message information (MI) to turn ON/OFF the anticipated power utilization.

## 4. Methodology

**Figure 2:**
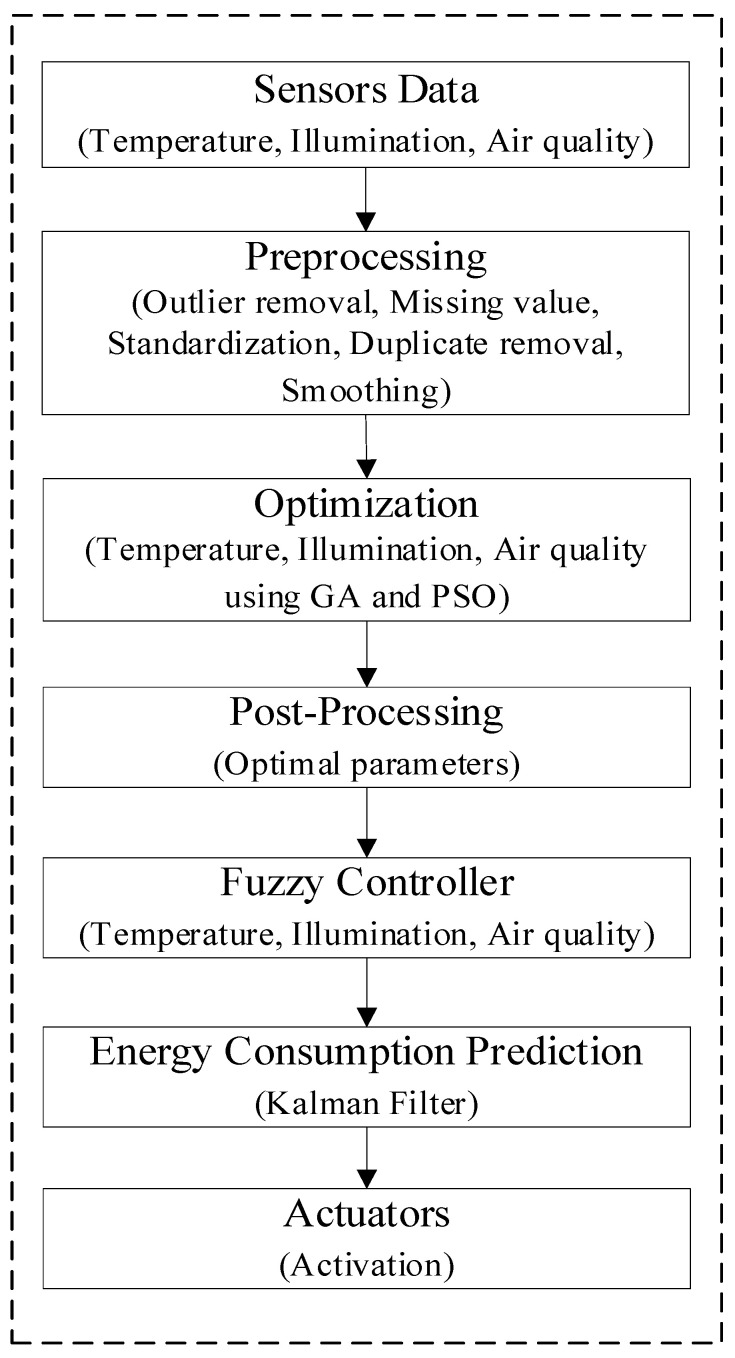
Flow chart of the proposed methodology.

Figure 2 illustrates the methodology flow chart of the proposed PMC model.

### 4.1. Multi-Processing

The multi-preprocessing is carried out for every primary module to preprocess the input data by employing a smoothing technique. The sensing information is additionally monitored to avoid the anomalies of outliers, empty cell information, abnormal structure of the information, and irregularity.

In case the information is discovered to be anomalous, it then merely eliminates the anomaly’s information, zero cell information, and brings the information into a standard structure. At the point when the sensing information becomes handled and in the standard structure, it is formerly forwarded to the next component of the framework called optimization.

After optimization, smoothing is once more carried out to smooth the OP; however, this time, provisional smoothing is carried out. Provisional smoothing indicates that, if the smoothing parameters result in a degraded OCI, those parameters are not nominees for smoothing, unless the parameters are smooth when it brings about improvement in the OCI. After smoothing the OP, the refreshed OP further improve the OCI. The inaccuracy covariance between the smooth real data and the refreshed OP is forwarded to the next component, called the fuzzy controller. The process of preprocessing/post-preparing is carried out for each part of the model until the data are ready to be offered over to the actuators.

### 4.2. Optimization

Steps for parameter optimizations and OCI based on PSO and GA [56].

The PSO steps for parameters’ optimizations and OCI are as follos:

- Initialization
  - Setting constants k max, c_1_, c_2_, r_1_, r_2_, w_0_
  - Random initialization of particle positions *x_i_ ∈ D* in *R^n^ for i* = 1…*p*
  - Random initialization of particle velocities
  - 0 ≤ v_0_^i^ ≤ v_0_^max^ for i = 1…p
  - Set k = 1
- Optimize
  - Evaluate *f_k_^i^* for particle *X ^i^_k_*
  - If *f ^i^_k_* ≤ *f ^i^_best,_ then f ^i^_best_ = f ^i^_k_, p^i^ = x ^i^_k_*
  - If *f ^i^_k_* ≤ *f ^g^_best,_ then f ^g^_best_ = f ^i^_k_, p^g^ = x^i^_k_*
  - Once the stopping norm is reached, then, move to step iii
  - Revise particle velocity vector *v^i^*_*k*+1_
  - Updating particle position vector *x^i^*_*k*+1_
  - Increasing *i* (index for particles). If *i* > pop, then, increase *k* (index for iterations), and after this, keep *i* = 1
  - Jump to 2 (i)
- Initialization
- Dismiss PSO optimization and obtain OP.

*V_i_* (*k* + 1) = *αV_i_* (*k*) + *m*_1_*r*_1_ [*P_best_*_(*i*)_(*k*)] + *m*_2_*r*_2_[*G_best_*(*k*) − *x_i_*(*k*)] (1)

*X_i_* (*k* + 1) = (*x_i_* (*k*) + *V_i_* (*k* + 1))/2 (2)

where α is the inertia weight, which is usually slightly smaller than 1, *m*_1_ and *m*_2_ are two positive constants, *r*_1,_ and *r*_2_ are two randomly generated numbers between [0, 1], P_best(i)(k)_ is the best previous position of the ith particle, G_best(k)_ is the best previous position of all particles in the swarm, and k is the iteration index.

GA phases for OCI and optimization:

1. A randomized initial population is defined
2. The objective function is calculated for the OCI using “(3)”
3. Select the best candidates based on the rank-based selection method.
4. ‘One point’ crossover is performed.
5. We obtain the off-springs after crossover.
6. Comfort for the off-springs is calculated.
7. Populations of steps (3) and (5) are combined.
8. Perform mutation, if mutation criteria meet.
9. The steps from 1 to 8 are frequently repeated up to the required number of iterations.
10. Select the best-fitted chromosome after the arrival of termination criteria.

The parameters are chosen by experimenting with the program for *λ* times to accomplish optimum output. The GA breaks when the maximum number of generations is met, or no substantial variation is detected in the fitness for *µ* generations. The maximum size of the population is 100. The single point crossover with 0.9 probability and 0.1 rate of mutation is selected. The GA stochastic operators (population size, crossover rate, and mutation rate) are set after running the GA for λ times. The investigations are accomplished using Intel(R) Core (TM)i3-2130 3.40 GHz with 8 GB RAM. The C # 2012 is used for the simulation. When the GA assessment procedure ends, the finest fitted individuals are selected to obtain the OPs and OCI.

### 4.3. Comfort

The user OCI can be determined using “(3)” [1]

*OCI* = *β*_1_ [1 − (*e_T_*/*T_set_*)^2^] + *β*_2_ [1 − (*e_L_*/*L_set_*)^2^] + *β*_3_ [1 − (*e_A_*/*A_set_*)^2^] (3)

where “OCI” is the targeted fitness function and the objective is to obtain the maximal value for this function. It designates the complete OCI, and it comprises illumination, temperature, and air quality. The OCI shifts somewhere in the range of ‘0’ and ‘1’, where ‘0’ signifies least or least OCI, and ‘1’ signifies the most noteworthy or greatest OCI. *β*_1_, *β*_2_ and *β*_3_ are the consumer distinct factors that settle any probable conflicts between the three occupant’s comfort factors. *β*_1_ + *β*_2_ + *β*_3_ = 1 at any time. In “(1)”, *eT* is the inaccuracy variance between the OPs of cutting-edge energy optimization (temperature in this state) and real sensor temperature. This is the least inaccuracy variance; the extreme will be the OCI. So, it can be deduced here that there is an opposite association between the OCI and inaccuracy variance of the parameters (T, L, A). As the inaccuracy variance is the input to the fuzzy regulator, which affirms the lesser inaccuracy variance, the lesser will be the CP. So, in this angle, the OCI has a converse relationship with power utilization. The lower the CP, the more elevated the OCI will be. So, the OCI relies on the inaccuracy variance for each of the (T, L A) set points. If the inaccuracy variance for each of the defined set points reduces, the OCI increases, and vice versa. This satisfies our essential design targets to limit the power utilization and to progress the OCI. *err_L_* is the inaccuracy variance for illumination between OP and real sensor illumination. *err_A_* is the inaccuracy variance for air quality between OP and real sensor air quality. *T_set_*, *L_set_*, and *A_set_* are the user set points of temperature, light, and air quality.

### 4.4. Coordinating Agent

The coordinating agent obtains the OPs of the building from the fuzzy controllers. The agent then updates the power requirements of the building based on existing energy and optimized required power to accomplish the OCI. The acclimated building power is matched with the required power to achieve the actual power utilization. The resultant power of the agent is then forwarded to the subsequent module known as prediction.

Equations (4)–(6) are available in [3], where total energy is *P* (*k*), which is equal to the sum of energy load used for illumination, temperature, and air quality. The representation of total available power sources is *P_available_*, and the maximum power that we are using for input to the building from the grid station or local micro power sources is indicated by *P_max_* (*k*).

*P_T_*_(*k*+1)_ = *P_T_*_(*k*)_ (4)

*P_L_*_(*k*+1)_ = *P_L_*_(*k*)_ (5)

*P_A_*_(*k*+1)_ = *P_A_*_(*k*)_ (6)

*P_T_*_(*k*)_ + *P_L_*_(*k*)_ + *P_A_*_(*k*)_ = *P_available_*_(*k*)_ (7)

*P_available_* ≤ *P_max_* (8)

### 4.5. Fuzzy Logic Controllers

A mathematician Lotfi Aliasker Zadeh, who is a well famous scientist at the California University at Berkley [57], introduced the theory of fuzziness.

The OP, real parameters, and the change in rate in the parameters can be given as input to the fuzzy logic. The power controllers provide output power based on the membership functions. The resultant output of the fuzzy-based controller(s) is the required energy to control the building lighting, temperature, and quality of air.

We set three controllers for temperature, lighting, and air quality. The input for the temperature controller is the error difference in optimized parameters and real smooth environmental parameters. Besides this, the error in the rate of change *ce_T_* in temperature is also considered as input to the temperature controller for efficiency. The fuzzy logic-based illumination, temperature, and air-quality controller’s rules and their corresponding input/output membership functions for each of the three controllers i-e illumination, temperature, and air-quality are presented in earlier work [2].

In “(9)”, “(10)” and “(11)” [3] γ_A_, γ_T_, and γ_L_ are the increment association with utilized energy (*P*) in time (*k*) for each of the air quality, temperature, and illumination. θ signifies the weight factor to balance the corresponding associations. The value of θ is in between [0, 1], while “*d*” represents the fundamental operation power of the ventilator.

*γ_T_* = *θ* × *P_T_*/*K* (9)

*γ_L_* = *θ* × *P_L_*/*K* (10)

*γ_A_* = *θ* × *P_A_*/*K* × *d* (11)

### 4.6. Kalman Filter

It is one of the best estimators, which can be used for time series data. Naturally, the Kalman filter is a recursive technique so that new quantities can be treated, as they are available to use. This filter tackled the overall problem of prediction by trying to predict the state (x ∈ Rn) of a discrete-time-controlled procedure, which is controlled by the standard linear stochastic difference equation. In “(12)” and “(13)”, the random normal distribution variables mt and nt denote the process and measurement noise, respectively.

The measurement noise covariance (*R*) and the process noise covariance (*Q*) matrices are altered with each time step. In our case, it is assumed to be constant. In “(12)”, the matrix (*A*) relates the state at the last time step (*t* − 1) to the state at the present step (t) in the lack of process noise. In general, the value of *A* may vary with each time step (*t*), but here, it is assumed as constant. The matrix (*P*) relates the optional control input to the state (*x*). The matrix (*H*) in “(13)” relates the state to the measurement (*Zt*). In general, the value of *H* may vary with each time step (*t*), but here, it is assumed as constant.

*Xt* = *Ax* (*t* − 1) + *P* + *m* (*t* − 1) (12)

*Zt* = *Hx* (*t*) + *nt* (13)

*m* ~ *N* (0, *Q*) (14)

*n* ~ *N* (0, *R*) (15)

### 4.7. Message Information (MI)

**Figure 3:**
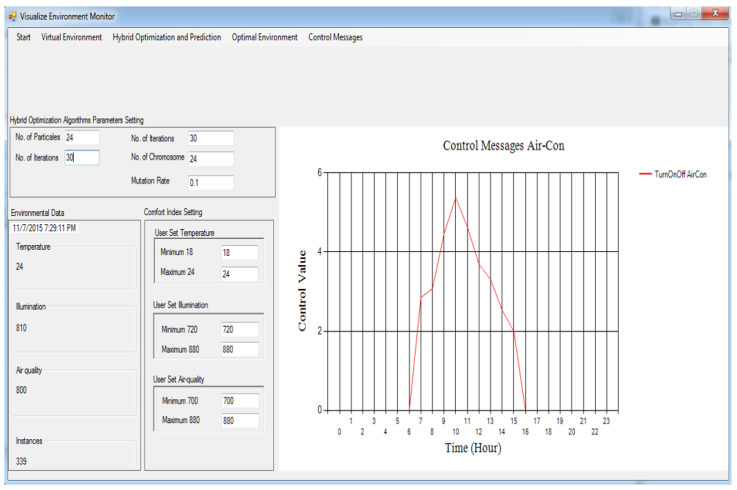
Control messages for air-con based on the proposed framework.

**Figure 4:**
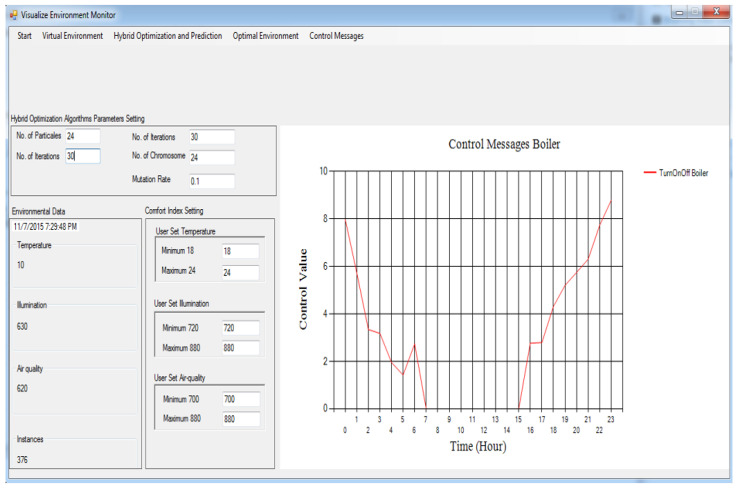
Control messages for boiler based on the proposed framework.

Figure 3 and Figure 4 show the message information (MI) to switch on/off the air-con and boiler correspondingly. Assuming the MI value is zero, it implies that the indoor temperature environment and optimal temperature are identical, and the respective actuator must be shut down. If the MI value is somewhere in the range of 0 and 3, a particular actuator will be switched on in a slow mood. Assuming the MI brings about a value somewhere in the range of 3 and 6, then, the actuator will be switched on in medium mood.

If the MI value is more than 6, then the actuator will be switched on at high speed. Figure 3 shows the MI to turn on/off the air-con. Here, we can see that the MI for air-con brings about a value more than zero between 6 h and 17 h, so the air-con for this situation is turned on during all levels between these hours, while for the remainder of the time, the air-con remains closed, including between 6 h and 16 h. In Figure 4, we can see that the kettle is turned on between 0 h and 7 h and between 15 h and 23 h. For the remainder of the time, it is turned off due to either the running of the air-con or the environmental parameters and optimal parameters remaining identical, which means that the indoor environment is comfortable enough.

**Figure 5:**
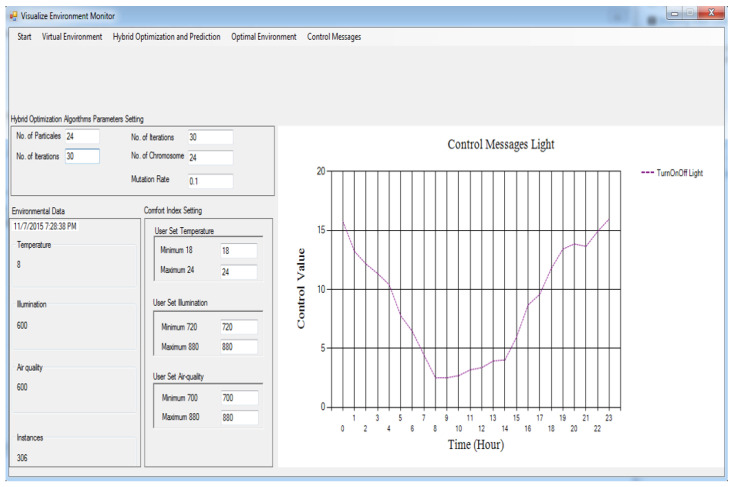
Control messages for light based on the proposed framework.

**Figure 6:**
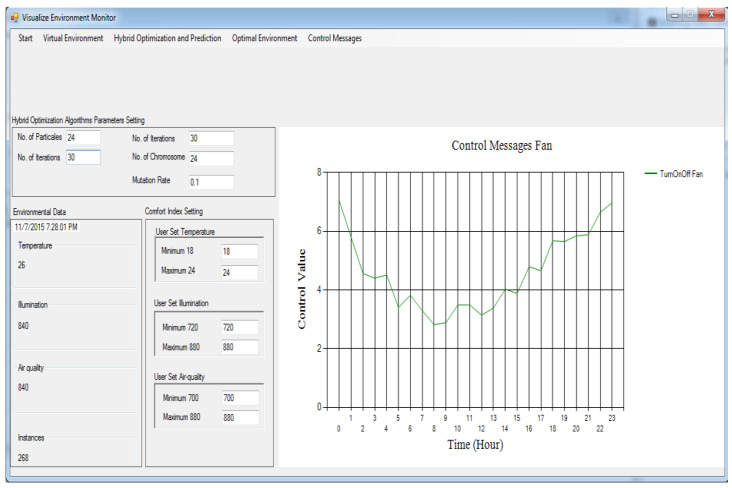
Control messages for fan based on the proposed framework.

Figure 5 and Figure 6 show the MIs to switch on/off the lights and fan correspondingly. For the light, we can see that the MI results in a value between 1 and 17, which implies that the light is switched on throughout 24 h. The light utilization diminishes as the daylight time shows up and upsurges as the daylight time wraps up. As far as the activation of the fan is concerned, in Figure 6, we can see that the MI results in a value more than zero, i.e., between 0 and 23 h, so the fan is switched on throughout 24 h.

### 4.8. Switching Regulator

The switching regulator oversees the existing power sources. For instance, if the outer energy source is unfit to transfer adequate energy to the building, or if the energy is more expensive, the framework will shift to the in-home power sources and vice versa.

### 4.9. Building Devices/Gadgets

The building gadgets are the devices which utilize the power in an indoor environment. The well-known devices used are AC, which is used to make the indoor environment cool, and the boiler, which is used to make the indoor atmosphere warm and cooler keeping palatable items at a suitable and ideal temperature. The sensor gadgets are utilized to record environmental information identified with temperature, illumination, and air quality. In the proposed framework, the actuators obtained the signals displayed in Figure 3, Figure 4, Figure 5, and Figure 6, respectively.

Every actuator has four levels: level 0, level 1, level 2, and level 3. Level 0 means turn off, level 1 means turn on at slow speed, level 2 means turn on actuator at medium speed, and level 3 means turn it on at high speed. Every emulator made the MI change its present status inside the residential building. At the point when these actuator emulators acquire the MI, the relevant state is altered to refresh the internal environmental conditions. The control signals characterized in Section 4.7 are utilized as MIs to trigger corresponding actuators at various levels.

## 5. Implementation Setup

Simulink and Matlab were used for input and output membership function formation.

The concrete simulation conceded in C# 2012. End-user preference set points range is Lset = [720, 880] (lux), Tset = [66, 78] (Kelvin), and Aset = [700, 880] (ppm).

## 6. Results

**Figure 7:**
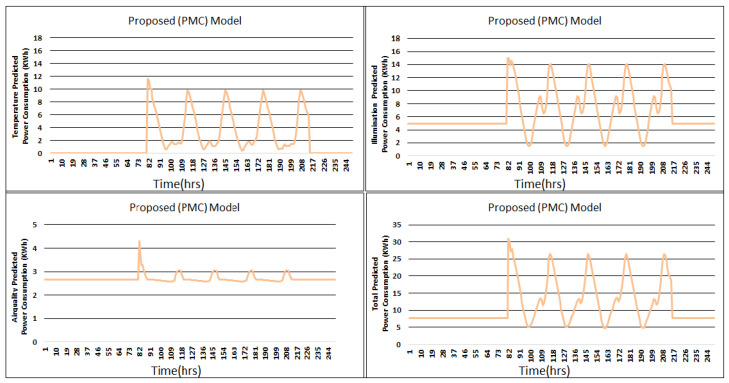
Proposed PMC framework predicted consumed power for temperature, illumination, air quality, and total power consumption.

Figure 7 shows the energy consumption prediction results of the proposed PMC model for the temperature, illumination, air quality, and total predicted power consumption. The graphs in Figure 7 show the power consumption trends during the hours of power disturbance. The consumed power in case of temperature, illumination, and air quality is 546.16 KWh, 1615.37 KWh, and 674.21, respectively.

**Figure 8:**
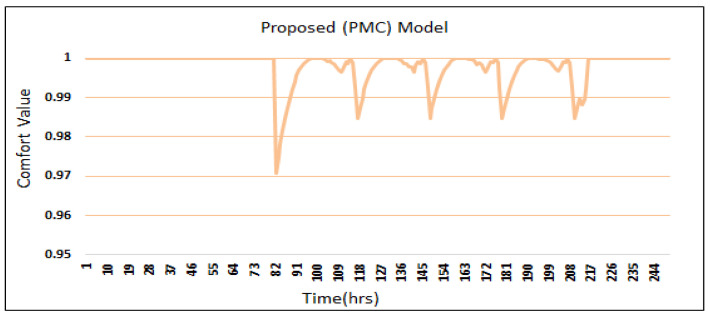
Comfort index of the proposed PMC frameworks.

Figure 8 shows the OCI of the proposed PMC model. First-time power disturbance occurs at 82 h; at this time, the PMC model’s OCI goes down to 0.97 but quickly recovers to 1. When the second power disturbance occurs, the PMC model’s OCI slightly goes down to 0.999. So, in all the power disturbances, the PMC model’s OCI recovers quickly to its maximum level (i.e., 1).

## 7. Discussion

**Figure 9:**
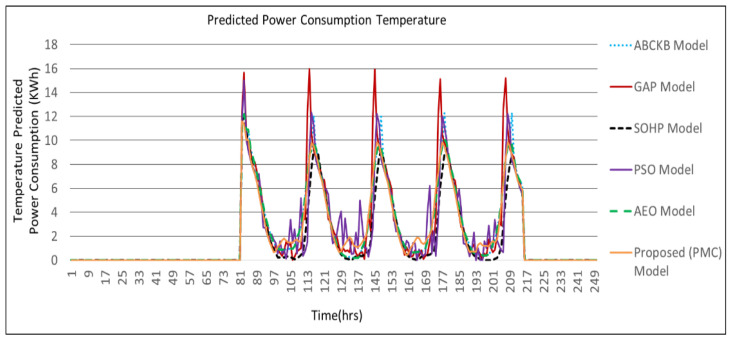
Proposed PMC framework based on predicted power consumption vs. ABCKB vs. GAP versus SOHP vs. PSO vs. AEO framework for temperature.

**Figure 10:**
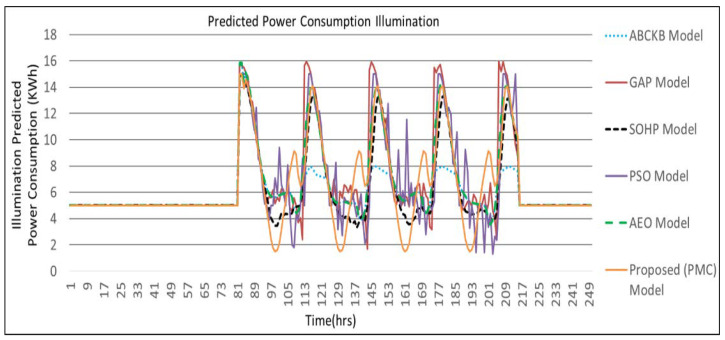
Proposed PMC framework based on predicted power consumption vs. ABCKB vs. GAP vs. SOHP vs. PSO vs. AEO model for illumination.

**Figure 11:**
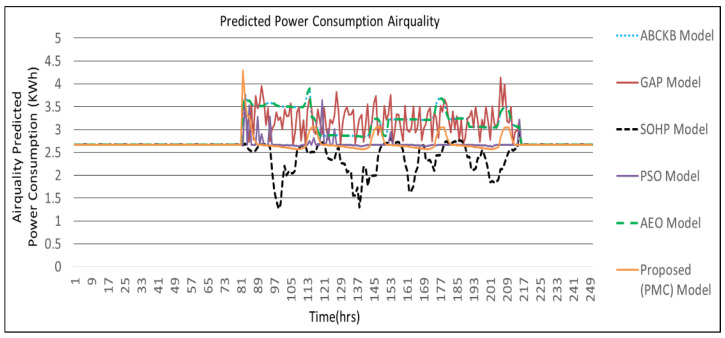
Proposed PMC framework based on predicted power consumption vs. ABCKB vs. GAP versus SOHP vs. PSO vs. AEO framework for air quality.

**Figure 12:**
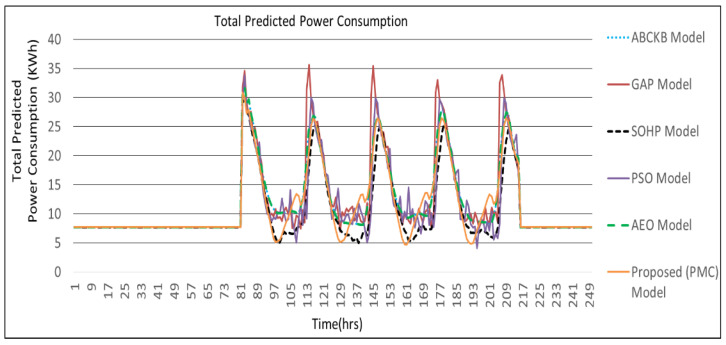
Proposed PMC framework based on total predicted power consumption vs. ABCKB vs. GAP vs. SOHP vs. PSO vs. AEO framework.

Figure 9, Figure 10, Figure 11 and Figure 12 describes the comparisons of energy consumption. The *X*-axis demonstrates the time in hours, while the *Y*-axis demonstrates the predicted energy consumption in kilowatts/hours. The OCIs between 0.0 and 1.0 show the minimum and maximum user OCI, correspondingly.

From the results of Figure 9 and Table 2, it can be observed that, in the case of power consumption for temperature, the PMC framework utilized a smaller amount of power as opposed to the GAP framework [3], PSO-based framework [5], and AEO framework [8]. As environmental disruption occurs, the proposed PMC framework utilizes a smaller amount of power compared to the GAP framework [3] and PSO framework [5] where no hybrid optimization and multi-preprocessing are involved. The optimized parameters and controllers achieve a lesser amount of power usage. Power consumption in the case of illumination is presented in Figure 10. The proposed PMC framework utilized the lowest power by comparing it with its corresponding GAP framework [3], PSO framework [5], and AEO framework [8]. Figure 11 presents the effect of power consumption in the case of air quality. Here, the PMC framework utilized the lowest power as compared to its corresponding GAP framework [3], PSO-based framework [5], and AEO framework [8]. Figure 12 demonstrates the total predicted power usage for the proposed PMC framework, GA-based power consumption prediction with no hybrid optimization and multi-preprocessing framework (GAP) [3], SOHP framework [4], PSO-based optimization framework [5], AEO framework [8], and ABCKB framework [9]. The total consumed power of the proposed PMC framework is much less than its counterpart models [3,5,8,9]. The proposed optimized PMC framework consumes a little bit more power as compared to the SOHP framework [4] but provides a much better OCI.

The power disorder first emerges at 82 h. The OCI of the proposed PMC model with prediction and multi-preprocessing goes down to 0.97, which is the same to GA based predicted framework GAP [3]. When the second power-unsettling disorder occurs, the GAP framework quickly goes down when contrasted with the proposed PMC framework. At 114 h, the proposed PMC framework tarnished to 0.999 when contrasted with 0.992 for the SOHP framework [4] and 0.974 for each of the GAP framework [3], PSO framework [5], and ABCKB framework [9].

Primarily, in all instances of power degradation, the proposed PMC framework offers an improved OCI when compared with existing methodologies such as the GAP [3], SOHP [4], PSO [5], ABCKB [9], and FA–GA frameworks [10] where no multi-preprocessing was applied. So, at whatever point there is an environmental augmentation, the proposed PMC framework recovers its OCI sooner than existing frameworks [3,4,5,8,9,10].

**Figure 13:**
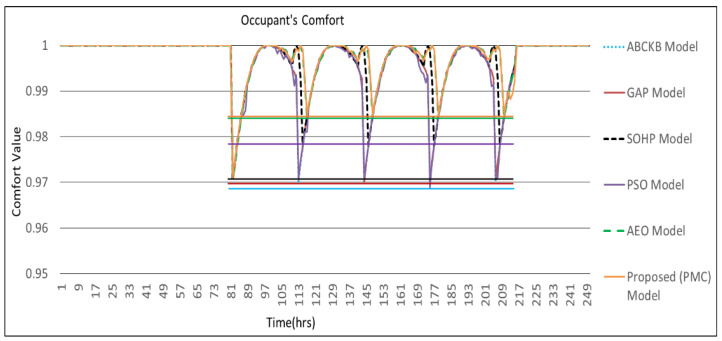
Comfort value comparisons of frameworks presented in [3,4,5,8,9,10] vs. proposed PMC framework.

Figure 13 shows the OCI results for the proposed PMC framework, ABCKB framework, GAP framework, SOHP framework, AEO framework, and PSO framework. The proposed PMC framework gives better and more conspicuous OCI when compared with existing frameworks [3,4,5,8,9,10]. The horizontal lines here demonstrate the baseline OCI provided by all frameworks during various intervals. However, in the proposed PMC model, less power is utilized in contrast with that of the GAP framework, PSO framework, and AEO framework, yet the proposed PMC model accomplished an extraordinary and better OCI. Of the existing frameworks, the SOHP framework is much better than the GAP framework, PSO framework, ABCKB framework, and FA–GA framework concerning OCI.

Table 2 clearly describes the claims of Figure 9, Figure 10, Figure 11 and Figure 12 quantitatively.

## 8. Conclusions

In this research paper, a hybrid power optimization and control methodology for OCI and energy conservation using intelligent algorithms GA and PSO in conjunction with multi-preprocessing called PMC is introduced. The paper tends to address both the energy effectiveness and OCI in a green building environment. To support the user’s contact with the framework, the user’s set points are considered in characterizing the OCI. The fundamental aim of the proposed model is to increase OCI according to user preferences and decline energy usage. The proposed PMC framework depends on hybrid energy optimization and multi-preprocessing together with the control of building actuators. The actuators of the building obtained message information from the proposed PMC framework and worked likewise. The proposed PMC framework utilized hybrid optimization based on GA and PSO techniques to optimize the environmental parameters and afterward predict power utilization using the Kalman filter. The parameters we improved are temperature, lightning, and air quality, which constitute the OCI in a smart environment.

The proposed PMC model is compared with recent methodologies like the GA-based forecast GAP framework [3], SOHP framework [4], PSO framework [5], AEO framework [8], ABCKB framework [9], and FA–GA framework [10]. The proposed PMC framework contributes unique and better OCI when compared with its partner’s GAP framework [3], SOHP framework [4], PSO framework [5], ABCKB framework [9], and FA–GA framework [10]. The proposed PMC framework additionally accomplished (greatest OCI as 1, average OCI as 0.99, and least OCI as 0.970) when compared with the existing FA–GA framework [10] (greatest OCI as 0.987, average OCI as 0.965, and least OCI as 0.957). The proposed PMC framework consumed substantially less power as compared with its partner GAP framework [3], PSO framework [5], and AEO framework [8]. The PMC framework gives practically the same OCI when compared with the AEO framework [8], however, consumes substantially less power than AEO. Using the proposed PMC power control framework for a smart environment, the building’s indoor environment can be made pleasant according to user-set points. The proposed PMC intelligent methodology is also confirmed through an emulator as compared to existing frameworks [3,4,5,9,10]. For real applications, the PMC framework can be incorporated with the SCADA software of buildings (https://www.scadaengine.com/, accessed on 11 July 2024).

In the future, we plan to further improve the occupant’s comfort index and minimize energy consumption by applying ensembles of other evolutionary techniques such as Ant Colony Optimization (ACO) with GA, and ACO with PSO.

## Figures and Tables

**Table 1:** Notations and their descriptions.

Symbol	Description	Symbol	Description
T	Temperature	K	Time
A	Air-quality	OCI	Occupant’s comfort index
L	Illumination	D	Process power for air quality
SCP	Smooth consumed power	P_max(k)_	Overall power provided by the outside or inside power sources
CP	Consumed power	OP	Optimal P
P_(k)_	Aggregated power	Ģ	Number of successive generations
RP	Required power	ϴ	Weight element
Ω	Total No. of generations	PCP	Predicted consumed power
e_T_	Inaccuracy variance in temperature	T_set_, L_set_, A_set_,	Parameters set by users
e_L_	Inaccuracy variance in illumination	P_available(k)_	Aggregated power resources (outside and inside)
e_A_	Inaccuracy variance in air quality	USP	User set points
ce_T_	Adjustment of error difference in temperature		

**Table 2:** Comparisons of energy consumption.

Model	Temperature	Illumination	Air Quality	Total Power Consumption
SOHP [4]	435.45	1542.32	623.44	2601.21
PSO [5]	544.26	1634.09	672	2850.57
ABCKB [9]	560.99	1491	742.52	2953.36
AEO [8]	551.41	1659.43	742.52	2953.36
GAP [3]	581.55	1704.91	739.79	3026.24
Proposed (PMC)	546.16	1615.37	674.21	2835.74

## Data Availability

The raw data supporting the conclusions of this article will be made available by the authors on request.
